# Hemodynamic Forces Regulate Cardiac Regeneration-Responsive Enhancer Activity during Ventricle Regeneration

**DOI:** 10.3390/ijms22083945

**Published:** 2021-04-11

**Authors:** Fang Geng, Jinmin Ma, Xueyu Li, Zhengyue Hu, Ruilin Zhang

**Affiliations:** 1 School of Life Sciences, Fudan University, Shanghai 200438, China; 18210700018@fudan.edu.cn (F.G.); 17110700042@fudan.edu.cn (J.M.); xueyuli17@fudan.edu.cn (X.L.); 18210700042@fudan.edu.cn (Z.H.); 2 School of Basic Medical Sciences, Wuhan University, Wuhan 430071, China

**Keywords:** heart regeneration, regeneration-responsive enhancer, hemodynamics, Notch signaling

## Abstract

Cardiac regenerative capacity varies widely among vertebrates. Zebrafish can robustly regenerate injured hearts and are excellent models to study the mechanisms of heart regeneration. Recent studies have shown that enhancers are able to respond to injury and regulate the regeneration process. However, the mechanisms to activate these regeneration-responsive enhancers (RREs) remain poorly understood. Here, we utilized transient and transgenic analysis combined with a larval zebrafish ventricle ablation model to explore the activation and regulation of a representative RRE. *lepb*-linked enhancer sequence (*LEN*) directed enhanced green fluorescent protein (EGFP) expression in response to larval ventricle regeneration and such activation was attenuated by hemodynamic force alteration and mechanosensation pathway modulation. Further analysis revealed that Notch signaling influenced the endocardial *LEN* activity as well as endogenous *lepb* expression. Altogether, our work has established zebrafish models for rapid characterization of cardiac RREs in vivo and provides novel insights on the regulation of *LEN* by hemodynamic forces and other signaling pathways during heart regeneration.

## 1. Introduction

Cardiac infarction is one of the leading causes of death worldwide, mainly due to the low proliferative capacity of human cardiomyocytes [1,2,3]. By contrast, zebrafish hearts can completely regenerate in several injury models [4,5,6,7]. Multiple genes and signaling pathways have been identified to be vital for zebrafish heart regeneration [5,8,9,10,11,12], yet most of them are conserved in other species as well [13,14,15,16]. This suggests that the key to the difference of regenerative capacity among species may not be attributable to specific genes but to their mechanism of activation, such as regulatory elements that play important roles in zebrafish heart regeneration.

Enhancers are key regulatory elements that control the spatiotemporal patterns of gene expression during development [17,18,19]. Enhancers activate gene transcription from target promoters, despite large variation in their distance and orientation, by engaging with transcription factors simultaneously [20,21,22,23]. Enhancers possess distinct chromatin structure and epigenetic markers [24,25], thus many techniques have been developed for enhancer prediction, such as ChIP-seq, ATAC-seq, DNase-seq, and FAIRE-seq [19,24,26,27,28]. However, functional characterization of enhancer activity in vivo is required to validate predicted enhancers, and zebrafish are ideal models for enhancer validation [29,30]. Furthermore, recent studies have revealed the involvement of enhancers in tissue regeneration [31,32,33,34,35,36,37,38]. For example, *leptin b* (*lepb*), one homologue of the mammalian *Leptin* gene which regulates energy homeostasis, is significantly induced during zebrafish tail fin and ventricle regeneration [31]. The *lepb*-linked regeneration enhancer (*LEN*) is identified through RNA-seq and H3K27Ac ChIP-seq, is termed the tissue regeneration enhancer element (TREE) and can be utilized to promote cardiomyocyte proliferation in infarcted mouse hearts [31]. The *inhba* enhancers from killifish (*K-IEN*) and zebrafish (*Z-IEN*), but not the enhancer from human (*H-IEN*), are regeneration-dependent in killifish resected hearts, suggesting that the change in regeneration-responsive enhancers (RREs) during evolution may be account for the loss of regenerative capacity [39]. Although multiple RREs have been reported, how injury signals activate them to execute regeneration program remains unclear [40,41].

In this report, we aimed to monitor the activity of a cardiac RRE (*LEN*) in real time and explore the mechanisms of its activation. We generated a *LEN*-directed transgenic reporter fish and examined enhancer activities in a larval zebrafish ventricle ablation model. We demonstrated that endogenous *lepb* expression increased in response to ventricle regeneration, and activation of *LEN* in the endocardial layer was attenuated by hemodynamic force alteration. Further analysis revealed that modulation of the mechanosensation pathway and Notch signaling influenced *LEN* activity. Overall, our work provides novel insights on the regulation of *LEN* by hemodynamic forces and other signaling pathways during heart regeneration and establishes a zebrafish model for rapid characterization of cardiac RREs in vivo.

## 2. Results

### 2.1. lepb Is Induced during Zebrafish Larval Ventricle Regeneration

**Figure 1:**
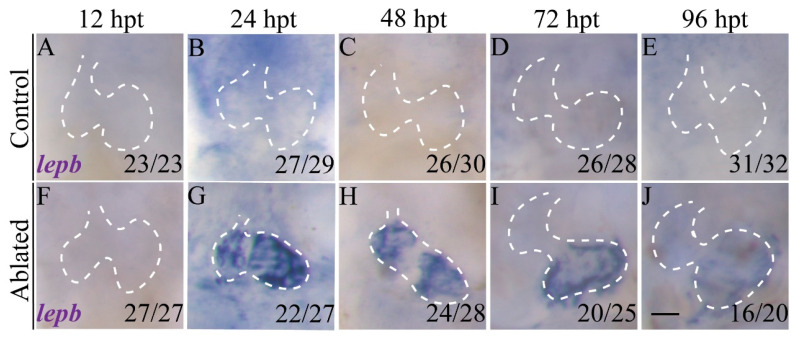
*lepb* is induced during zebrafish larval ventricle regeneration. (**A**–**J**) Whole-mount in situ hybridizations showed that *lepb* expression was upregulated in ablated hearts of *Tg(vmhc:mCherry-NTR)* fish compared to control hearts. Scale bar, 25 μm. Dashed lines outline the hearts. Numbers indicate the ratio of representative staining observed. hpt, hours post treatment. NTR, nitroreductase.

*lepb* expression is highly induced in the adult zebrafish amputated hearts [31]. We previously generated a larval ventricle regeneration model using a genetic cardiac ablation line, *Tg(vmhc:mCherry-NTR)* fish expressing nitroreductase (NTR), in ventricular cardiomyocytes driven by the *ventricular myosin heavy chain* (*vmhc*) promoter, where NTR can convert the prodrug metronidazole (MTZ) into a toxic metabolite causing cell death [5]. To characterize *lepb* expression during larval ventricle regeneration, we treated *Tg(vmhc:mCherry-NTR)* fish with MTZ at 3 days post fertilization (dpf) and performed whole-mount in situ hybridization (WISH) at multiple stages. *lepb* expression was undetectable in control hearts at all time points and in ablated hearts at 12 h post treatment (hpt) (Figure 1A–F). At 24 hpt, *lepb* expression was sharply induced in the atrium and ventricle of ablated hearts and slightly decreased at 48 hpt (Figure 1G,H). Notably, *lepb* was not expressed in the atrioventricular canal (AVC). At 72 and 96 hpt, *lepb* expression in the ventricle significantly decreased (Figure 1I,J).

### 2.2. LEN Is Activated in the Endocardium during Ventricle Regeneration

**Figure 2:**
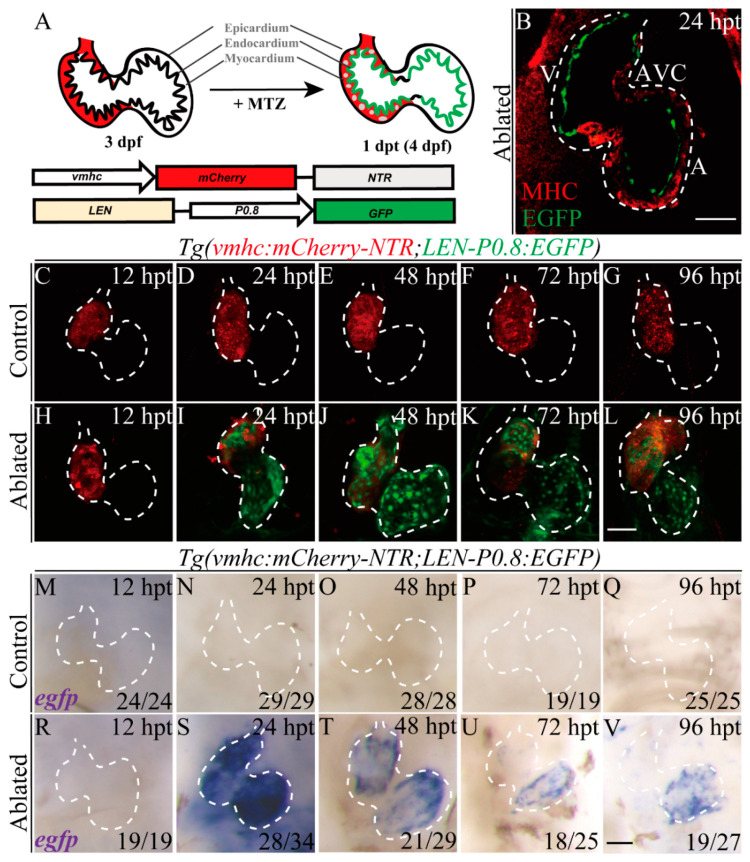
*LEN* is activated in the endocardium during ventricle regeneration. (**A**) Schematic diagrams of the transgenic constructs and ventricular ablation process of *Tg(vmhc:mCherry-NTR; LEN-P0.8:EGFP)* fish. (**B**) Confocal optical section image showed enhancer-directed fluorescence (green) in the endocardium of ablated *Tg(vmhc:mCherry-NTR; LEN-P0.8:EGFP)* hearts at 24 hpt. Red, MHC immunostaining by MF20. A, atrium; AVC, atrioventricular canal; MHC, myosin heavy chain; V, ventricle. (**C**–**L**) Confocal stack projections showed enhancer-directed fluorescence (green) dramatically increased in ablated hearts of *Tg(vmhc:mCherry-NTR; LEN-P0.8:EGFP)* fish compared to control hearts. (**M**–**V**) Whole-mount in situ hybridizations showed dynamic *egfp* expression in ablated hearts of *Tg(vmhc:mCherry-NTR; LEN-P0.8:EGFP)* compared to control hearts. Scale bars, (**B**–**L**) 50 μm, (**M**–**V**) 25 μm. Dashed lines outline the hearts. Numbers indicate the ratio of representative staining observed. dpf, days post fertilization; hpt, hours post treatment.

To examine whether *LEN*, as with adult ventricle apex amputation [31], exhibits regeneration-responsive activity during larval ventricle regeneration, we generated a stable transgenic line *Tg(LEN-P0.8:EGFP)* containing *LEN* and 0.8 kb upstream sequences of *lepb (P0.8)* fused with enhanced green fluorescent protein (EGFP), and crossed it with *Tg(vmhc:mCherry-NTR)* (Figure 2A). No EGFP signal was observed in control hearts during development (Figure 2C–G). After ventricle ablation, *LEN*-directed EGFP signal was strongly induced in the hearts at 24 hpt and remained, though slightly decreased, through 96 hpt when most ablated hearts recovered (Figure 2H–L). The *LEN*-directed EGFP signal was expressed in the endocardium of the atrium and ventricle but not in the AVC (Figure 2B). Since EGFP fluorescence is relatively stable and may not reflect the dynamic activity of the enhancer [5], we also performed WISH to examine *egfp* mRNA expression (Figure 2M–V). The results revealed that *egfp* expression dramatically increased in ablated hearts at 24 hpt and gradually reduced through 96 hpt, resembling the endogenous expression pattern of *lepb* and suggesting that *LEN* activity is transient during larval ventricle regeneration. Thus, we confirmed that *LEN* showed cardiac regeneration-responsive activity upon ablation of the larval ventricle. The reporter line *Tg(vmhc:mCherry-NTR; LEN-P0.8:EGFP)* was established successfully, which could be used to further explore the activation mechanisms of RREs.

### 2.3. Mutation of Predicted AP-1 Binding Site Has No Effect on LEN Activity

**Figure 3:**
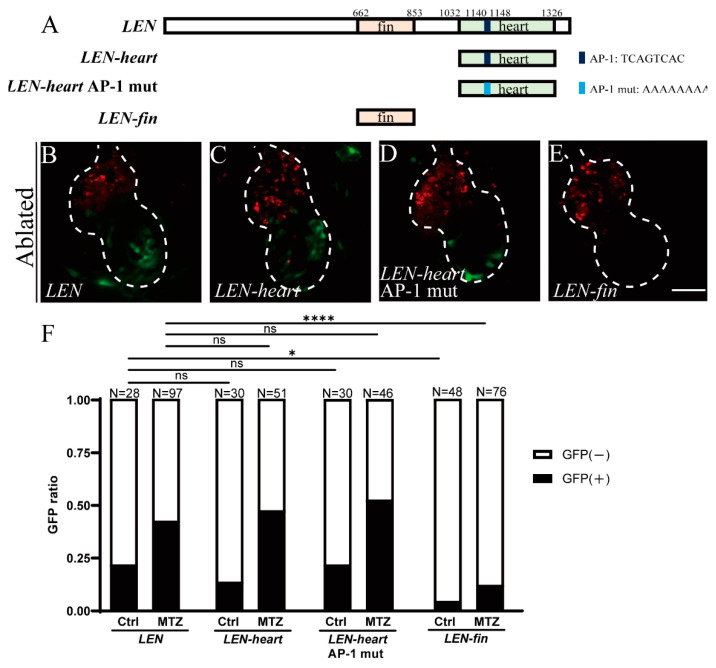
Transient analysis of enhancer activity for *LEN* motifs. (**A**) Schematic diagrams of full-length *LEN, LEN-heart, LEN-heart* with AP-1 mutation and *LEN-fin* elements. The original and mutated sequences of the predicted AP-1 binding site are also indicated. (**B**–**E**) Confocal stack projections of ablated hearts at 24 hpt from larvae injected with *LEN* (**B**), *LEN-heart* (**C**), *LEN-heart AP-1 mut* (**D**) and *LEN-fin* (**E**) constructs. (**F**) Quantification of the EGFP ratio of control and ablated hearts in *LEN, LEN-heart, LEN-heart AP-1 mut* and *LEN-fin* injected groups at 24 hpt. N = 28, 97, 30, 51, 30, 46, 48, 76, respectively. Chi-Square Test, ns, not significant, *, *p* < 0.05, ****, *p* < 0.0001. Scale bar, 50 μm. Dashed lines outline the hearts. hpt, hours post treatment.

*LEN* contains two motifs which are responsible for its activity in tail fin and adult heart regeneration, respectively [31] (Figure 3A). To validate *LEN-heart* activity in larval ventricle regeneration, we performed transient analysis of enhancer activity for *LEN*, *LEN-heart* and *LEN-fin*. Although there were a few larvae expressing sporadic EGFP signals in control groups due to mosaicism in the transient assay, the percentages of larvae expressing cardiac EGFP signal after ablation dramatically increased in the *LEN* group (42%, N = 97) and in the *LEN-heart* group (47%, N = 51). However, this induction was greatly reduced in the *LEN-fin* group (12%, N = 76) (Figure 3B,C,E,F). The results affirmed that *LEN-heart* had stronger RRE activity than *LEN-fin* in larval ventricle regeneration.

Recent studies have revealed that activator protein 1 (AP-1) binding sites are enriched in RREs and *K-IEN* activity is dependent on the presence of the AP-1 binding site, which suggests that AP-1 may be essential for regeneration responses [39,42]. There is a predicted AP-1 binding sequence (TCAGTCAC) in the *LEN-heart* motif (Figure 3A). To explore its function, we generated a construct of *LEN-heart* with a mutated sequence (AAAAAAAA) and performed transient analysis. The result indicated this sequence may not be required for *LEN-heart* activity since the mutated construct *LEN-heart AP-1 mut* could still direct EGFP expression in ventricle regeneration (52%, N = 46) in a pattern similar to *LEN-heart* (Figure 3C,D,F). It is of great interest to further explore the mechanisms of *LEN* activation.

### 2.4. Activation of LEN Is Attenuated by Hemodynamic Force Alteration

**Figure 4:**
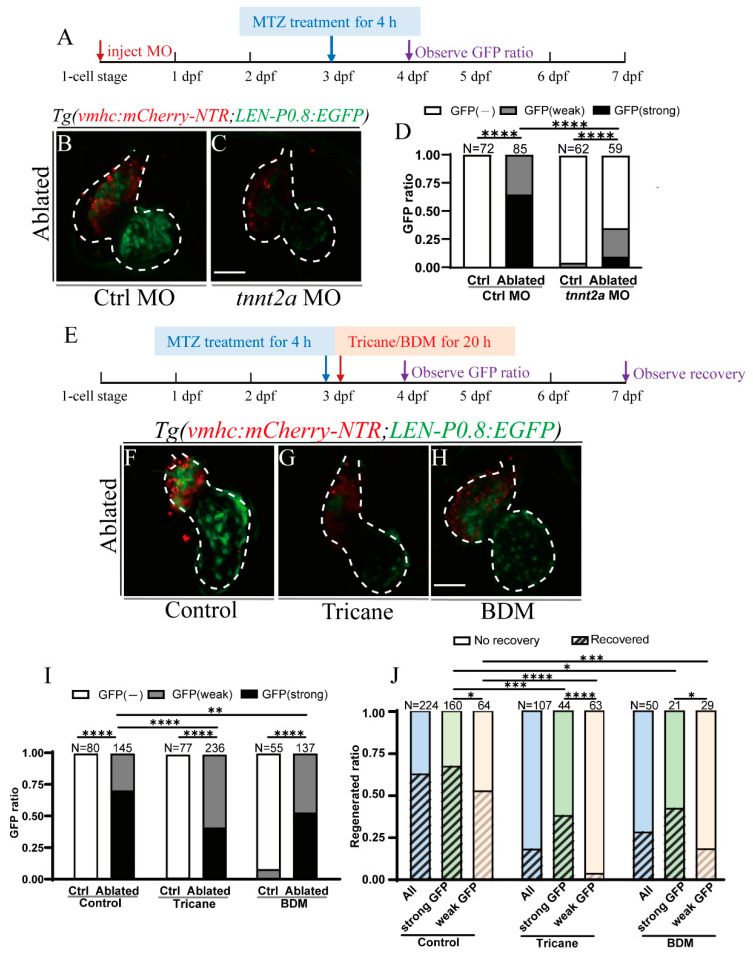
Activation of LEN is attenuated by hemodynamic force alteration. (**A**) Schematic timeline diagram of MO injection and MTZ treatment. (**B**,**C**) Confocal stack projections of ablated *Tg(vmhc:mCherry-NTR; LEN-P0.8:EGFP)* hearts in Ctrl MO (**B**) and *tnnt2a* MO (**C**) injected groups at 24 hpt. (**D**) Quantification of the EGFP ratio of control and ablated hearts in Ctrl MO and *tnnt2a* MO injected groups at 24 hpt. *N* = 72, 85, 62, 59, respectively. Fisher’s Exact Test (2X3), ****, *p* < 0.0001. (**E**) Schematic timeline diagram of MTZ treatment and hemodynamic alteration. (**F**–**H**) Confocal stack projections of ablated *Tg(vmhc:mCherry-NTR; LEN-P0.8:EGFP)* hearts in Control (**F**), Tricaine (**G**), and BDM (**H**) treated groups at 24 hpt. (**I**) Quantification of the EGFP ratio of control and ablated hearts in control, Tricaine, and BDM treated groups at 24 hpt. N = 80, 145, 77, 236, 55, 137, respectively. Fisher’s Exact Test (2X3), **, *p* < 0.01, ****, *p* < 0.0001. (**J**) Quantification of the regeneration ratio of ablated hearts in control, Tricaine, and BDM treated groups at 96 hpt. N = 224 (160 strong + 64 weak EGFP signal), 107 (44 + 63), 50 (21 + 29), respectively. Chi-Square Test, *, *p* < 0.05, ***, *p* < 0.001, ****, *p* < 0.0001. Scale bars, 50 μm. Dashed lines outline the hearts. dpf, days post fertilization; hpt, hours post treatment; MO, morpholino; MTZ, metronidazole.

We previously showed that hemodynamic force alteration played crucial roles in the activation of endocardial Notch signaling and the regulation of zebrafish ventricle regeneration [43,44]. To investigate whether hemodynamic forces also have an impact on endocardial *LEN* activation, we injected *tnnt2a* morpholino (MO) [45] at one-cell stage which knocked down cardiac troponin T expression and abolished heart contraction and blood flow (Figure 4A). At 24 hpt, the EGFP signal in ablated hearts was strongly reduced in the *tnnt2a* MO-injected group compared to the control MO (Ctrl)-injected group (Figure 4B,C). In Ctrl morphant, there was no EGFP fluorescence without ablation (N = 72); however, all ablated hearts (N = 85) showed cardiac EGFP signal, among them 65% of hearts showed strong EGFP expression and 35% showed weak EGFP expression. In *tnnt2a* morphant, the proportion of *LEN*-directed EGFP signal after ablation decreased sharply to 36% (N = 59), with only 10% showing strong EGFP expression (Figure 4D). 

Tricaine and 2,3-Butanedione monoxime (BDM) are two muscle relaxants used for blocking cardiac contractility to alter hemodynamic forces [43,44]. As *tnnt2a* knockdown may influence zebrafish development and cardiac contractility besides blocking blood flow [46], we also used Tricaine and BDM to temporarily reduce blood flow after heart ablation to further confirm the influence of hemodynamic forces on LEN activity. Ablated larvae were immersed with 6 mM Tricaine or 7 mM BDM from 4 to 24 hpt (Figure 4E). Ablated hearts in the Tricaine and BDM treated groups showed weaker EGFP expression than that in the ablated control group (Figure 4F–H). Quantification showed that the percentage of larvae expressing strong cardiac EGFP signal after blood flow inhibition dropped to 42% in the Tricaine-treated ablated group (*N* = 236) and 53% in the BDM-treated ablated group (*N* = 137), compared to 70% in the ablated control group (*N* = 145) (Figure 4I). Next, we examined the regeneration ratios of all larvae, larvae expressing strong EGFP signal and larvae expressing weak EGFP signal. The results showed that the regeneration ratio in the control treated group (63%, *N* = 224) was higher than that in the Tricaine treated group (19%, *N* = 107) and the BDM treated group (29%, *N* = 50), and the regeneration ratios of larvae expressing strong or weak EGFP signal showed a similar trend (Figure 4J). We also analyzed the regeneration ratio of larvae expressing strong and weak EGFP signal in each ablated group. In the ablated control group, 67% of larvae showing strong cardiac EGFP expression (N = 160) fully regenerated the injured hearts, whereas the regeneration ratio in larvae showing weak EGFP expression dropped to 53% (*N* = 64). In the Tricaine-treated ablated group, the regeneration ratios of larvae expressing strong and weak EGFP signal were 39% (*N* = 44) and 5% (*N* = 63), respectively. In the BDM-treated ablated group, the corresponding regeneration ratios were 43% (*N* = 21) and 19% (*N* = 29). Thus, larvae expressing strong EGFP signal exhibited better heart recovery than larvae expressing weak EGFP signal in all three ablated groups (Figure 4J), which indicated that the enhancer activity may be relevant to the heart regeneration ratio. 

### 2.5. Primary Cilia and Mechanosensitive Ion Channel Affect LEN Activity

**Figure 5:**
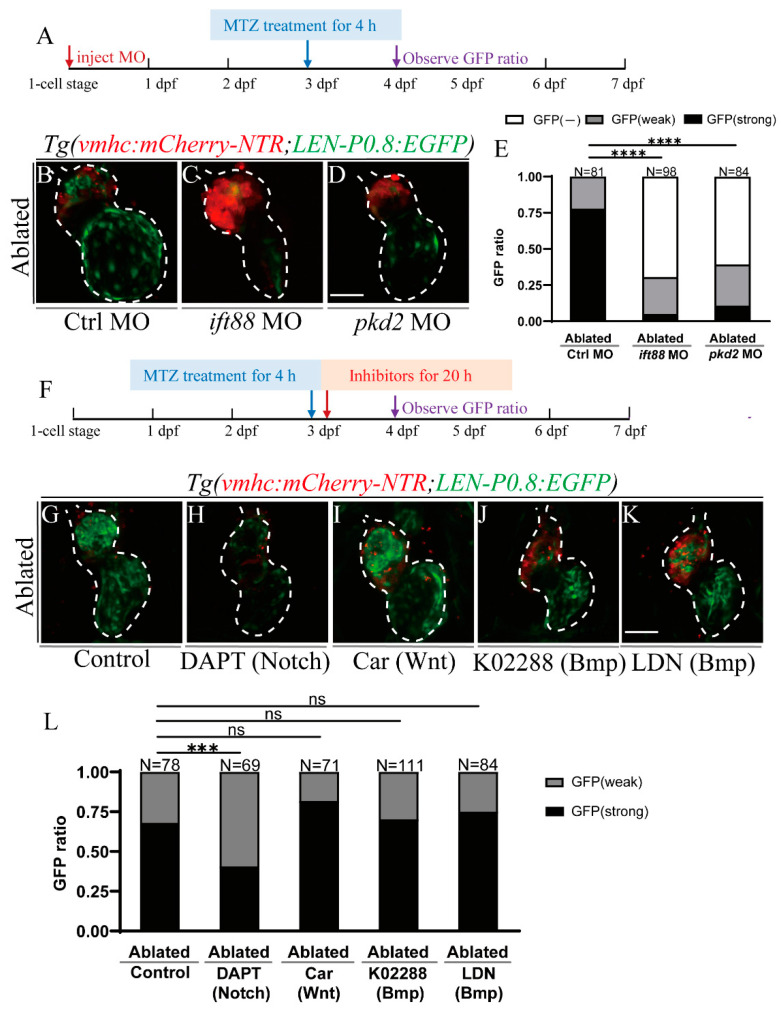
Primary cilia, mechanosensitive ion channel, and Notch signaling influence *LEN* activity. (**A**) Schematic timeline diagram of MO injection and MTZ treatment. (**B**–**D**) Confocal stack projections of ablated *Tg(vmhc:mCherry-NTR; LEN-P0.8:EGFP)* hearts in Ctrl MO (**B**), *ift88* MO (**C**) and *pkd2* MO (**D**) injected groups at 24 hpt. (**E**) Quantification of EGFP ratio of ablated hearts in Ctrl MO, *ift88* MO and *pkd2* MO injected groups at 24 hpt. N = 81, 98, 84, respectively. Fisher’s Exact Test (2X3), ****, *p* < 0.0001. (**F**) Schematic timeline diagram of MTZ treatment and signaling pathway inhibition. (**G**–**K**) Confocal stack projections of ablated *Tg(vmhc:mCherry-NTR; LEN-P0.8:EGFP)* hearts in control (**G**), DAPT (**H**), Car (**I**), K02288 (**J**) and LDN (**K**) treated groups at 24 hpt. (**L**) Quantification of EGFP ratio of ablated hearts in control, DAPT, Car, K02288 and LDN treated groups at 24 hpt. N = 78, 69, 71, 111, 84, respectively. Fisher’s Exact Test (2X3), ns, not significant, ***, *p* < 0.001. Scale bars, 50 μm. Dashed lines outline the hearts. dpf, days post fertilization; hpt, hours post treatment; Car, Cardiomogen-1; LDN, LDN193189; MO, morpholino; MTZ, metronidazole.

Considering that *LEN* activation was affected by hemodynamic alteration, we then aimed to investigate the influences of other factors involved in the mechanical sensation and transmission of hemodynamic forces. Previous studies demonstrated that the primary cilia and mechanosensitive ion channels function as mechanical sensors for shear stress to regulate zebrafish heart development and regeneration [43,44,47]. Ift88 is a transport protein that is essential for cilia assembly [48] and Pkd2 is a permeable transient receptor ion channel activated by shear stress [49]. We injected *ift88* and *pkd2* MOs at one-cell stage to knock down these genes (Figure 5A). EGFP expression was significantly reduced in ablated hearts of *ift88* and *pkd2* morphants compared to that in ablated hearts of control morphants at 24 hpt (Figure 5B–D). The ratio of larvae expressing strong cardiac EGFP signal dramatically decreased in ablated *ift88* morphants (5%, *N* = 98) and *pkd2* morphants (11%, *N* = 84) compared to control morphants (78%, *N* = 81) (Figure 5E). These results confirmed that components of the mechanosensation pathway, such as the primary cilia and mechanosensitive ion channel, affect *LEN* activity during ventricle regeneration.

### 2.6. Notch Signaling Influences LEN Activity

Recent studies have shown that reduced blood flow and cilia knockdown affect endocardial Notch activation during cardiac regeneration [43,44]. To investigate whether Notch signaling is involved in *LEN* activation, ablated larvae were treated with a potent pharmacologic inhibitor for Notch signaling [5,43], (3,5-Difluorophenacetyl)-l-alanyl-S-phenylglycine-2-butyl Ester (DAPT), for 20 h (Figure 5F). *LEN*-directed EGFP expression after DAPT treatment was much weaker than that in the ablated control group. Quantification showed that the ratio of strong EGFP signal in the DAPT-treated ablated group was 41% (*N* = 69) compared to 68% in the ablated control group (*N* = 78) (Figure 5G,H,L). Notch signaling facilitates zebrafish heart regeneration through promoting Wnt antagonists [50]. Inhibition of Wnt signaling by Cardiomogen-1 [51] increased the ratio of strong EGFP expression in the ablated group to 82% (*N* = 71), though this was not statistically significant (Figure 5I,L). 

Multiple signaling pathways, besides Notch and Wnt signaling, were activated during zebrafish heart regeneration [7,9]. Our larval ventricle ablation model provided a convenient way to rapidly examine their involvement in RRE activation in vivo. We utilized small molecules to modulate three important pathways, Bmp, RA, and EGF signaling, to further explore the mechanisms of *LEN* activation (Figure 5F). Bmp signaling is an indispensable regulator of zebrafish heart regeneration [44,52]. K02288 is a selective type I Bmp receptor inhibitor, mainly targeting ALK1, ALK2, and ALK6 [53,54], while LDN193189 is a selective Bmp signaling inhibitor targeting ALK2 and ALK3 [55]. Inhibiting Bmp signaling did not affect *LEN* activity (Figure 5J–L). RA synthesized by endocardial and epicardial cells represents an early cardiac injury response [56]. EGF signaling is required for normal cardiovascular development in zebrafish [57]. Chemical modulation of these two signaling mechanisms also had no effect on *LEN* activity (Figure S1).

### 2.7. Inhibition of Blood Flow and Notch Signaling Attenuates lepb Induction during Ventricle Regeneration

**Figure 6:**
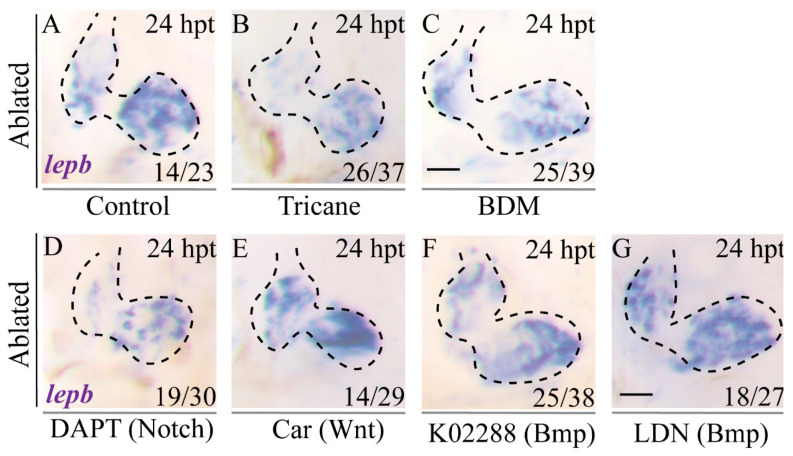
Inhibition of blood flow and Notch signaling attenuates *lepb* induction during ventricle regeneration. (**A**–**G**) Whole-mount in situ hybridizations showed that *lepb* expression in ablated hearts of *Tg(vmhc:mCherry-NTR) fish* at 24 hpt (**A**) was reduced in the Tricaine- and BDM-treated groups (**B**,**C**) and in the DAPT-treated group (**D**), slightly enhanced in the Car-treated group (**E**), and remained unchanged in the K02288- and LDN-treated groups (**F**,**G**). Scale bars, 25 μm. Dashed lines outline the hearts. Numbers indicate the ratio of representative staining observed. hpt, hours post treatment; Car, Cardiomogen-1; LDN, LDN193189.

To further validate the effect of blood flow and Notch signaling, we used WISH to examine *lepb* expression during ventricle regeneration upon different treatments. The results showed that *lepb* expression in ablated hearts at 24 hpt was downregulated in Tricaine and BDM treated groups compared to that in the control group (Figure 6A–C). *lepb* expression was significantly weakened in the DAPT-treated ablated group, slightly enhanced in the Cardiomogen-1-treated ablated group, and remained unchanged upon treatment with two Bmpr inhibitors (Figure 6D–G). Overall, these results suggested that endogenous *lepb* expression closely resembled *LEN* activity during ventricle regeneration and further affirmed that hemodynamic forces and Notch signaling were involved in *LEN* activation.

## 3. Discussion

Besides the traditional roles in controlling spatiotemporal gene expression during development, the new roles of enhancers in regulating tissue regeneration have recently become more appreciated [40,41,58,59]. *LEN* activity increases in response to both zebrafish tail fin and ventricle apex amputation [31]. Genome-wide replacement histone profiling reveals H3.3-enriched elements as potential RREs in heart regeneration [35]. *careg*, an upstream sequence of *ctgfa*, reveals a common regulation of regeneration in the zebrafish myocardium and fin induced by TGFβ/Activin-β signaling [32]. *K-IEN* element knockout disrupts fin and heart regeneration in killifish [39]. RREs have been identified in mammals as well. In mice, an enhancer element is shown to control *Bmp5* expression after bone fracture, skin, or lung injury but could not activate *Bmp5* during development [37]. In rats, a c-Jun-bound enhancer has been identified in the *Runx2* locus after nerve injury [33]. However, how these RREs are activated or whether they share common regulatory mechanisms remains largely unknown. In this study, we utilized a larval zebrafish ventricle ablation model to monitor the activity of a cardiac RRE in real time and explore the mechanisms of its activation. We confirmed the activity of *LEN* in response to ventricle regeneration resembling the endogenous *lepb* expression. Furthermore, we can dynamically observe the effect of genetic and pharmacological manipulation on RRE activation in vivo. Thus, our model provides a convenient way to compare and dissect the regulatory mechanisms of cardiac RREs and their functions in heart regeneration.

Hemodynamic forces are crucial for heart development and regeneration [60]. Goddard et al. showed that hemodynamic forces play an essential role in heart valve development through shear-responsive transcription factor KLF2 [61]. During zebrafish heart regeneration, hemodynamic alteration caused by heart injury can be sensed by endocardial cells through mechanosensitive ion channel Trpv4 and transduced to activate Notch signaling [44]. Li et al. also revealed that hemodynamic alteration activates Notch signaling through primary cilia and flow responsive Klf2a/Klf2b factors [43]. In the current study, we have shown that *LEN* was activated in the endocardium of ablated hearts and its activity was related with hemodynamic forces. Blocking blood flow through *tnnt2a* knock down or Tricaine/BDM treatment significantly reduced enhancer-directed EGFP fluorescence. Disruption of primary cilia-associated protein Ift88 or mechanosensitive channel Pkd2 also dramatically affected the activities of *LEN*. Our results suggest that mechanical sensation and transmission of shear stress may be regulatory mechanisms for cardiac RRE activation. It is worth noting that Ift88 may affect cardiac development through Hippo signaling [62] and Pkd2 could influence cardiac contraction through calcium signaling [63]; thus, the mechanisms underpinning their regulation of *LEN* activity warrant further exploration.

Notch signaling is activated by hemodynamic forces [43,44] and it interacts with multiple signaling pathways, including the Wnt, BMP and Nrg1/ErbB2 pathways, to promote zebrafish cardiac regeneration [44,50]. Our work illustrated that inhibition of Notch signaling by DAPT treatment reduced *LEN* activity. Previous studies showed that Notch reporter signals were first induced in endocardial cells around the AVC at 12 hpt and then expanded to the endocardium of the chamber [43]. However, EGFP expression directed by *LEN* was in the endocardium of the ventricle and atrium, except for AVC, and started from 24 hpt. Thus, our observation raises more unanswered questions. Do hemodynamic forces independently regulate *LEN* activity, or does it occur through Notch signaling? Are the effects of Notch signaling cell autonomous or cell non-autonomous? Why is *LEN* activity excluded from the AVC endocardium? All these questions remain to be further explored. 

Taken together, the models and methodology established in this study will be of great benefit for the future identification and characterization of new cardiac RREs. We also provide novel insights on how hemodynamic forces and signaling pathways modulate *LEN* activity, which lays a foundation for future studies on RRE activation.

## 4. Materials and Methods

### 4.1. Zebrafish Husbandry

Wild-type (AB) strain and transgenic zebrafish were raised and maintained using standard methods [64]. Embryos over 24 hpf were maintained in E3 water with 0.003% 1-phenyl-2-thiourea (PTU, Sigma, St. Louis, MO, USA) to prevent pigmentation. All experiments were performed in accordance with institutional and national animal welfare guidelines.

### 4.2. Generation of Transgenic Zebrafish Lines

The construct to generate transgenic lines was created by cloning a 0.8 kb *lepb* promoter sequence (*P0.8*) [31] into the pT2-mx-EGFP plasmid and *LEN* was then cloned upstream of the *P0.8* promoter. The PCR primers used to generate enhancer sequences are listed in Table S1. The sequences of *LEN-heart* and *LEN-heart AP-1 mut* were synthesized. Tol2 transposase mRNA was in vitro transcribed from pCS-TP plasmid using the mMessage mMachine T7 kit (Thermo Fisher Scientific, Waltham, MA, USA) [65]. Twenty pg transgenic construct was co-injected with 100 pg Tol2 transposase mRNA into one-cell stage embryos. Positive founders were identified through genotyping of F1 offspring. 

### 4.3. Ventricle Ablation

To perform ventricular cardiomyocyte ablation, 72 hpf larvae of *Tg(vmhc:mCherry-NTR)* outcrossed with different enhancer reporter lines were treated with 6 mM MTZ (metronidazole, Sigma, St. Louis, MO, USA) or 0.2% DMSO (dimethyl sulfoxide, Thermo Fisher Scientific, Waltham, MA, USA) for 4 h as previously described [5]. Treated larvae were washed with three changes of fresh E3 water at the end of ablation. Fluorescence was observed and imaged at indicated time points using the Leica M205FA fluorescence stereo microscope and the Zeiss LSM880 confocal microscope.

### 4.4. Morpholino Injection

Morpholino injections were performed as previously described [43]. Morpholinos were purchased from GeneTools (Philomath, OR, USA), dissolved in ddH_2_O containing 10% phenol red, and injected into one-cell stage embryos. The injection amounts per embryo were 1 ng for *tnnt2a* MO [66], 5 ng for *ift88* MO [67], and 10 ng for *pkd2* MO [68]. The injection amount of control MO was the same as those of the experimental groups. The MO sequences were listed in Table S1. All injected embryos were used for indicating experiments at specific stages.

### 4.5. Drug Treatment

To influence blood flow, control or ablated *Tg(vmhc:mCherry-NTR)* larvae were incubated in E3 water with 1.8 mM Tricaine (3-aminobenzoic acid ethyl ester, Sigma, St. Louis, MO, USA) or 10 mM BDM (2,3-butanedione monoxime, Sigma, St. Louis, MO, USA) directly after ablation for 15 h [43]. For signaling pathway studies, zebrafish larvae were incubated in E3 water with different drugs or solvent right after ablation for 20 h. A quantity of 100 μM of DAPT (Sigma, St. Louis, MO, USA) was used to inhibit Notch signaling [5]; 5 μM Cardiomogen-1 (Sigma, St. Louis, MO, USA) was used to inhibit Wnt signaling [51]; 7.5 μM K02288 (Selleck, Houston, TX, USA) or 5 μM LDN193189 (Selleck, Houston, TX, USA) was used to inhibit Bmp signaling [53,55]; 1 μM RA (Sigma, St. Louis, MO, USA) was used to activate RA signaling [69]; 5 μM PD153035 (Selleck, Houston, TX, USA) was used to inhibit EGF signaling [70]. 

### 4.6. In Situ Hybridization

Whole mount in situ hybridization was performed as previously described [5]. Primers used for *lepb*, *egfp* antisense probe synthesis were listed in Table S1. 

### 4.7. Immunofluorescence

Immunofluorescence staining on whole mount larvae was performed as previously described [5]. The primary antibody used was MF20 (DSHB, Iowa City, IA, USA), and the secondary antibody used was Alexa Fluor 555 goat anti-mouse IgG (Thermo Fisher Scientific, Waltham, MA, USA). Fluorescence images were obtained using a Zeiss LSM880 confocal microscope.

### 4.8. Quantification and Statistical Analysis

Larvae were divided into three groups with strong, weak or no EGFP expression in the hearts and the EGFP ratio was calculated as the number of larvae in each group over the number of total larvae. The heart regeneration ratio was calculated as the number of larvae with fully recovered hearts over the total number of ablated larvae at 96 hpt. Sample sizes were chosen on the basis of previous publications and verified by power analysis using R software, and are indicated in each figure legend. *p* values were obtained by Fisher’s Exact Test (2X3) or Chi-Square Test using R software. The statistical significance was set at *, *p* < 0.05, **, *p* < 0.01, ***, *p* < 0.001, and ****, *p* < 0.0001.

## Supplementary Materials

The following are available online at https://www.mdpi.com/article/10.3390/ijms22083945/s1, Figure S1: RA and EGF signaling do not affect LEN activity, Table S1: List of primers and morpholinos used in this study.
