# Supplementary material for: Hemodynamic Forces Regulate Cardiac Regeneration-Responsive Enhancer Activity during Ventricle Regeneration
Source: Int J Mol Sci. 2021 Apr 11;22(8):3945. doi: 10.3390/ijms22083945 (PMC8070559; doi:10.3390/ijms22083945)
Supplement: Supplementary file 1 [file ijms-22-03945-s001.pdf]

Supplementary materials

Supplementary Figure S1. RA and EGF signaling do not affect *LEN* activity

Supplementary Table S1. List of primers and morpholinos used in this study

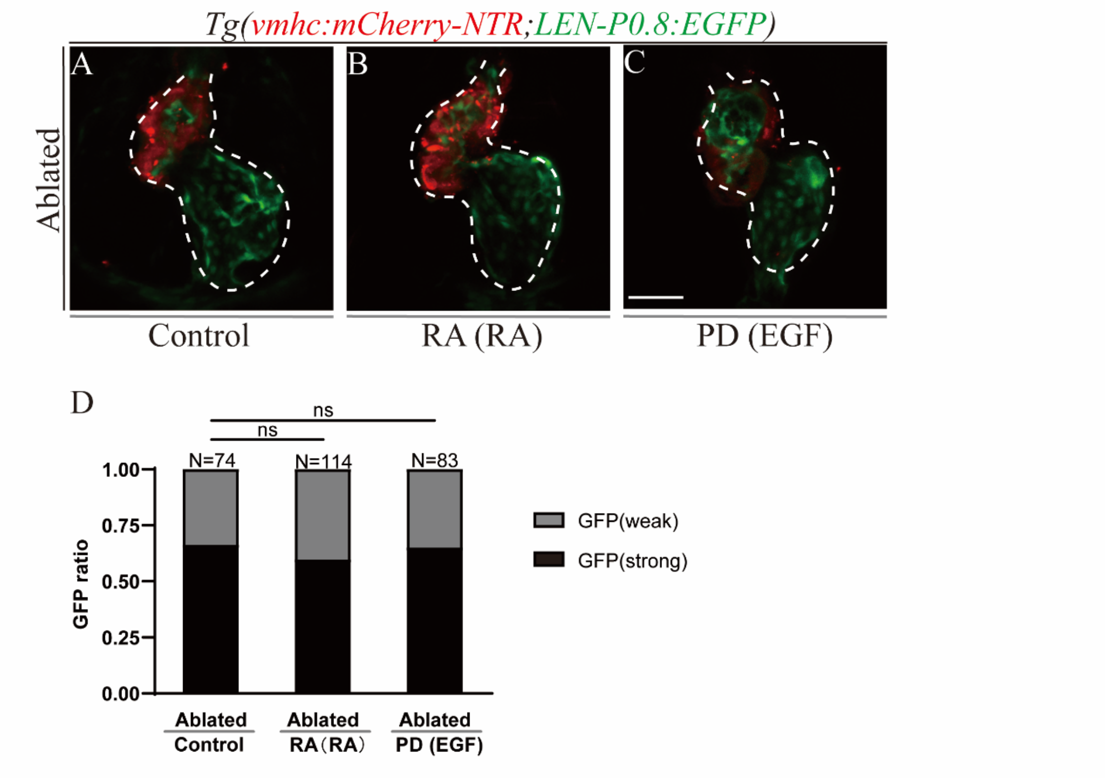

Supplementary Figure S1. RA and EGF signaling do not affect *LEN* activity

(A-C) Confocal stack projections of ablated *Tg(vmhc:mCherry-NTR; LEN-P0.8:EGFP)* hearts in control (A), RA (B), and PD (C) treated groups at 24 hpt. **(D)** Quantification of EGFP ratio of ablated *Tg(vmhc:mCherry-NTR; LEN-P0.8:EGFP)* hearts in control, RA and PD treated groups at 24 hpt. N=74, 114, 83, respectively. Fisher's Exact Test (2X3), ns, not significant.

Scale bar, 50 µm. Dashed lines outline the hearts. hpt, hours post treatment; PD, PD153035.

Supplementary Table S1. List of primers and morpholinos used in this study

Application	Primers	Sequences (5'-3')
Constructs	*P0.8*-F	ttcgggcttgttgaaagggt
	P0.8-R	tttcatttggtcttttgcagaaat
	LEN-F	actcgccaatttgcttctgtttc
	LEN-R	tggcatacacagcaaacatcatg
	LEN-fin-F	tcttatttttcagcattgtccttc
	LEN-fin-R	tgggaaacgcagcaatttcc
In situ hybridization	*lepb*-F	gctcccgaagacaggatacg
	lepb-R	gccacatctgtatctttgcagt
	egfp-F	atggtgagcaagggcgag
	egfp-R	ttacttgtacagctcgtccatgc
Morpholino	*tnnt2a* MO	CATGTTTGCTCTGATCTGACACGCA
	ift88 MO	CTGGGACAAGATGCACATTCTCCAT
	pkd2 MO	AGGACGAACGCGACTGGAGCTCATC
	control MO	CCTCTTACCTCAGTTACAATTTATA
